# Effect of Porosity and Pore Size on the Axial Compressive Properties of Recycled Aggregate Concrete

**DOI:** 10.3390/ma18122830

**Published:** 2025-06-16

**Authors:** Chunqi Zhu, Eryu Zhu, Bin Wang, Jiacheng Li, Tong Yao, Zhu Zhang

**Affiliations:** 1 School of Civil Engineering, Beijing Jiaotong University, Beijing 100044, China; 20115007@bjtu.edu.cn (C.Z.);; 2 School of Environment and Safety Engineering, North University of China, Taiyuan 030051, China

**Keywords:** initial pore, recycled aggregate concrete, mechanical property, gray correlation analysis, damage variable

## Abstract

Pores of different sizes and quantities are formed during the molding process of recycled aggregate concrete (RAC). However, few studies have examined the individual and combined effects of porosity and mesoscale pore size (pore size) on the axial compressive mechanical properties of RAC. In this study, the influence of porosity and pore size on the axial compressive mechanical behavior of RAC was examined by incorporating expanded polystyrene (EPS) particles to create prefabrication of pores. Additionally, crack development influenced by pores was analyzed using high-energy X-ray computed tomography (CT). Gray correlation analysis was employed to quantify the influence of pore size and porosity on compressive mechanical parameters. Furthermore, the combined effects of pore characteristics were assessed by introducing damage variables. It was shown that the compressive strength, strength reduction, elastic modulus, and modulus reduction exhibited linear correlations with porosity and exponential correlations with pore size. Cracks within the specimen predominantly propagate through the pores or along their edges. The influence of porosity on both strength and elastic modulus is more substantial than that of pore size. Moreover, the deterioration in mechanical properties is more pronounced when small pore size is coupled with high porosity, compared to the combination of large pore size and low porosity.

## 1. Introduction

Recycled aggregates (RAs) are obtained from construction and demolition waste (CDW) through crushing, washing, and screening processes [1]. The survey data indicate that replacing natural aggregates (NAs) with recycled aggregates (RAs) in engineering projects offers significant advantages in resource utilization and environmental protection [2]. For example, RAs produced from CDW can reduce non-renewable energy consumption by 58% and greenhouse gas emissions by 65% compared to NAs [3]. Moreover, long-term costs can be reduced by 40% [4]. Additionally, using RAs for pavement construction can result in cost savings of approximately 0.5 GBP/m^2^ [5]. RAC, as a typical heterogeneous porous material, can develop internal pores due to the air introduced during the mixing process and the evaporation of water during molding [6]. Factors such as construction techniques, vibration levels, and maintenance conditions can also contribute to the formation of pores [7,8,9]. Jivkov et al. [10] employed X-ray computed tomography to generate three-dimensional reconstructions of concrete, revealing a significant number of pores at both micro- and meso-scales. The presence of these pores inevitably has an impact on the mechanical properties [11,12] and durability [13,14] of concrete materials.

In recent years, relevant studies have been conducted to investigate the impact of pores properties on the mechanical properties of concrete. However, both experimental and numerical simulation studies have primarily focused on natural aggregate concrete (NAC) systems. The existing literature [15,16,17] indicates that due to differences in material systems, significant variations exist in the quantitative relationships between pore parameters and mechanical performance across different types of concrete. Therefore, whether the conclusions established for NAC can be directly applied to RAC still requires further validation.

Since pores mostly exist at the meso-scale, meso-scale numerical simulations have become a widely adopted method among researchers. Most of these studies regard concrete as a four-phase composite material, comprising aggregate, mortar, interfacial transition zone, and pores, and analyze the influence of pores on the mechanical properties of concrete materials by establishing a meso-scale model [18]. For example, Zhang et al. [19] demonstrated that aggregate content and porosity significantly influence the axial compressive properties of concrete and that the rate of crack propagation within the specimen is inverse to porosity. Wang et al. [20] investigated the influence of the spatial and size distribution of circular-like stochastic pores on the fracture behavior of concrete based on multiple fractal theory, finding that these stochastic circular pores induce distinct splitting tensile crack patterns. Zhang et al. [21] embedded zero-thickness cohesive elements into the finite element model and assigned them to the traction-detachment criterion to analyze the influences of aggregate volume content and porosity on the axial compressive parameters and the fracture process of concrete. Peng et al. [22] investigated the fracture behavior of notched beams and found that the distribution of pores and aggregates can induce crack bifurcation. The extent of bifurcation is closely related to the aggregate interface, aggregate composition, and pore distribution. Wang et al. [23] constructed circular and elliptical pores and analyzed the influences of pore distribution and volume content on axial tensile fracture properties, finding that porosity can significantly reduce the axial tensile strength of specimens. These studies have revealed the significant role of porosity in determining mechanical properties. However, most of the work has focused solely on the individual effects of porosity, with insufficient attention paid to pore size and the combined effects of pore size and porosity. Moreover, most of these studies have remained at a qualitative level in analyzing how pore structure influences mechanical properties. However, in reality, hardened concrete contains pores of varying sizes and quantities [24]. Porosity and pore size, as independent yet interrelated parameters, exert a coupled influence on the mechanical behavior of materials that cannot be overlooked. In addition, these mesoscale simulation studies have primarily focused on NAC, while there remains a significant lack of mesoscale simulation research on the mechanisms by which pore structure influences RAC. The primary reason for this gap is the scarcity of experimental data required to support the establishment and validation of such simulations. In summary, there is a clear need for quantitative experimental research to elucidate the influence of pores on the mechanical properties and failure modes of RAC.

To address these issues, this study achieved quantitative control of the initial porosity and pore size by adjusting the particle size and mass of EPS particles. Specimens with four porosity levels (2%, 4%, 6%, and 8%) and five pore size ranges (0.3–0.5 mm, 0.5–1 mm, 1–2 mm, 2–3 mm, and 3–5 mm) were prepared and subjected to axial compression tests to quantitatively analyze the influence of porosity and pore size on axial compressive performance. Additionally, using a self-developed high-energy X-ray CT, the development of cracks in RAC containing initial defects after compression was investigated, revealing the influence of pores on crack propagation at the mesoscale. Furthermore, grey correlation theory was employed to compare and evaluate the relative influence of porosity and pore size on the axial compressive strength and elastic modulus of RAC. Finally, damage variables were introduced to reveal the coupled effects of porosity and pore size. The research results not only provide experimental evidence for the validity of the mesoscale simulation analysis of RAC with defects but also offer important references for the design and engineering applications of RAC materials.

## 2. Materials and Methods

This study aims to examine the effect of porosity and pore size on the macroscopic axial compression mechanical properties of RAC. Porosity values ranging from 0–8% were considered, with four commonly used levels (2%, 4%, 6%, and 8%) selected from existing studies [16]. It is worth noting that inherent pores are inevitably present in RAC. Chen et al. [25] reported that the porosity of micrometer-sized pores in RAC is approximately 2.3%. Based on this value and the designed porosity introduced, the expected total porosity of the specimens was estimated. The expected total porosities corresponding to design porosities of 2%, 4%, 6%, and 8% were approximately 4.3%, 6.3%, 8.3%, and 10.3%, respectively. Skarżyński et al. [26] investigated the distribution of pores in three cross-sections of concrete specimens using high-precision X-ray computed tomography. The results showed that the pore diameters ranged from 0–4 mm, where the pores with a diameter between 0.25–2.03 mm accounted for approximately 75% of the total pore area. Based on this, in this paper, a pore size of 0.3–5 mm was chosen, with five distinct pore size categories: 0.3–0.5 mm, 0.5–1 mm, 1–2 mm, 2–3 mm, and 3–5 mm. Six specimens were prepared for each set of test conditions [27], totaling 126 specimens. In accordance with the Chinese standard GB/T 50081-2019 [28], the maximum and minimum values were excluded from each set of six data points, and the average of the remaining four data points was calculated to reduce the influence of extreme values on the experimental results, thereby ensuring the reliability of the data.

### 2.1. Materials and Mix Design

All coarse aggregates used were RAs, with particle sizes ranging from 5–25 mm. Table 1 shows the physical properties of RAs. The fine aggregate was natural river sand, and the water was tap water. The P.C 42.5 compound silicate cement was used, and all the cement properties comply with the Chinese standard GB175-2023 [29]. Polycarboxylic acid-based liquid high-performance water reducer was used. According to the recommended dosage range provided in the product manual (0.3–1.0% by mass of the cementitious materials), an initial dosage of 0.3% was selected. The dosage was subsequently increased in increments of 0.04% until the target slump range of 120 ± 10 mm was reached. The final dosage of the water reducer was determined to be 0.42%, which resulted in a measured slump of 124 mm. The mix design for the baseline RAC is shown in Table 2. This reference mix contains no EPS particles and has a porosity of 0%. It serves as the baseline for comparative analyses with samples of varying porosities and pore sizes, aiming to assess the impact of pore parameters on mechanical properties.

### 2.2. Fabrication and Testing of Specimens

**Figure 1:**
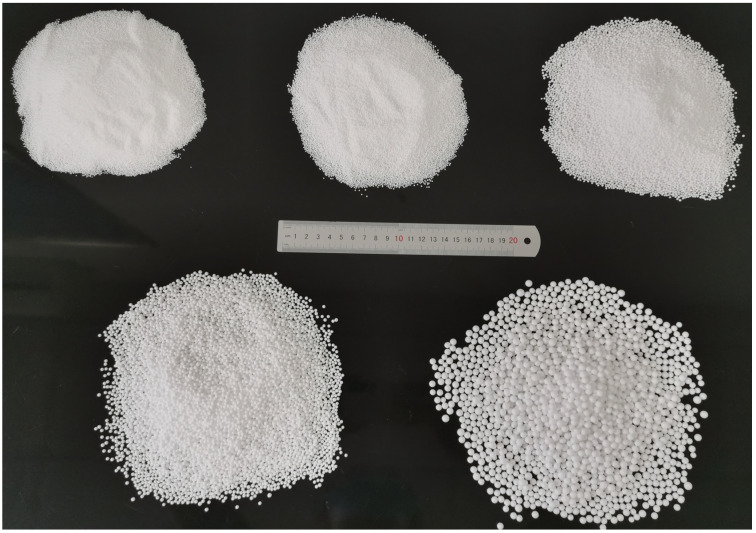
EPS particles of different sizes.

Currently, two commonly used methods are employed to create initial defects within concrete specimens: the incorporation of air-entraining agents [30] during mixing and the addition of EPS particles [31] to simulate initial pores. However, it is difficult to control the content and size of the pores introduced by using air-entraining agents [32]. To quantitatively analyze the effect of pores on the mechanical properties of RAC, initial pores were prefabricated using EPS particles in this paper. The EPS particles used are shown in Figure 1. They are composed of a stable polymeric foam derived from polystyrene and exhibit pronounced surface hydrophobicity and low density, with an internal porosity exceeding 95%. Their intrinsic mechanical strength is negligible. They were factory-produced and categorized into five particle size ranges: 0.3–0.5 mm, 0.5–1 mm, 1–2 mm, 2–3 mm, and 3–5 mm. For quantitative analysis and graphical representation, the median value of each range (i.e., approximately 0.4 mm, 0.75 mm, 1.5 mm, 2.5 mm, and 4.0 mm) was used as the representative particle size.

The mass of EPS particles required for each condition was calculated using Equation (1). Due to the low density of EPS particles, an electronic balance with an accuracy of 0.0001 g and a glass beaker were used to weigh the EPS particles. The weighed EPS particles were then placed into a plastic bag for storage and use. It is important to note that the “porosity” in this study refers to the designed porosity, which is artificially created by incorporating different masses of EPS particles. This designed porosity does not account for the inherent porosity of RAC. Therefore, the actual total porosity of RAC is higher than the designed porosity specified in this study.

(1) $$f_{\mathrm{pore}}=\frac{m_{\mathrm{pore}}}{V_{c}\rho_{\mathrm{pore}}}\times 100\%$$

where $m_{\mathrm{pore}}$ is the mass of EPS particles, kg; $V_{c}$ is the volume of concrete, m^3^; and $\rho_{\mathrm{pore}}$ is the density of EPS particles, kg/m^3^.

To make the EPS particles better dispersed inside the RAC, the EPS particles were first pretreated. Some sand was wetted with water and then mixed with EPS particles so that it could be uniformly wrapped in the wet sand. Meanwhile, the method of making cement mortar first was used to utilize the cohesive property of the slurry to encapsulate the EPS particles [33], thus preventing excessive uplift of the EPS particles.

The scheme of sample preparation was as follows.

(1) Prepare the required water and fine sand for the specimen. Take a portion of the fine sand and wet it with water. Add all the EPS particles to the wet sand and mix thoroughly to ensure that the EPS particles are evenly coated by the wet sand.
(2) Add the remaining sand and cement sequentially to the concrete mixer and dry mix for 1.5 min.
(3) Add 60% of the water and all of the water-reducing agent to the concrete mixer, then mix for 1.5 min to form a slurry-like cement mortar.
(4) Add the mixture of EPS particles and wet sand from the first step to the concrete mixer, and mix for 1.5 min.
(5) Add RAs and the remaining water to the concrete mixer, and mix for 3 min.

**Figure 2:**
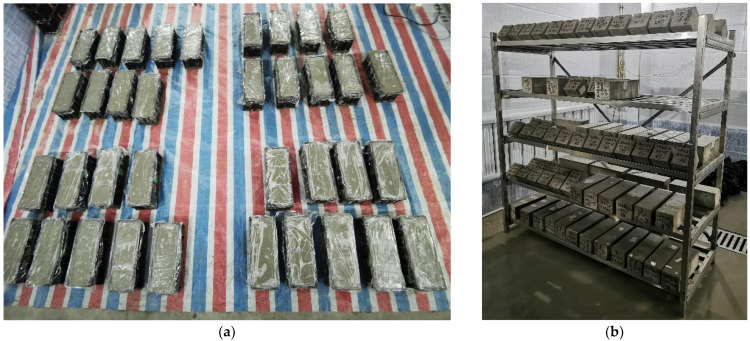
The process of specimen production: (**a**) specimens covered with cling film; (**b**) curing of specimens.

Then, the mixture was poured into the plastic mold with the dimensions of 100 mm × 100 mm × 300 mm and vibrated on a concrete magnetic vibration table for 20~22 s. The EPS particles vibrated on the surface were scraped back into the mold using a spatula. The surface of the specimen was then gradually leveled by tapping around the mold with a rubber mallet. Afterward, the specimen’s surface was covered with a layer of cling film (Figure 2a). The specimens were unmolded after being placed in the laboratory environment for 24 h. Subsequently, the unmolded specimens were placed in the standard curing room for curing (Figure 2b).

**Figure 3:**
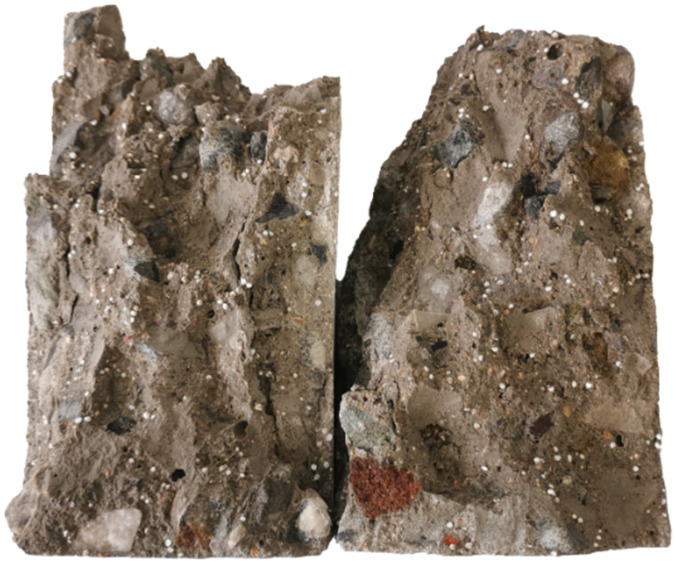
Dispersion of EPS particles inside the RAC specimen.

To verify the effectiveness of this process, a portion of the specimen was broken. To enhance the visibility of the EPS particles, the broken surface was wetted with water (Figure 3). It can be observed that the EPS particles within the sample exhibit a dispersed pattern with no obvious aggregation, closely resembling the naturally formed “initial pore” state. However, this observation is primarily based on qualitative judgment and has not yet been quantified using image analysis or porosity measurements.

**Figure 4:**
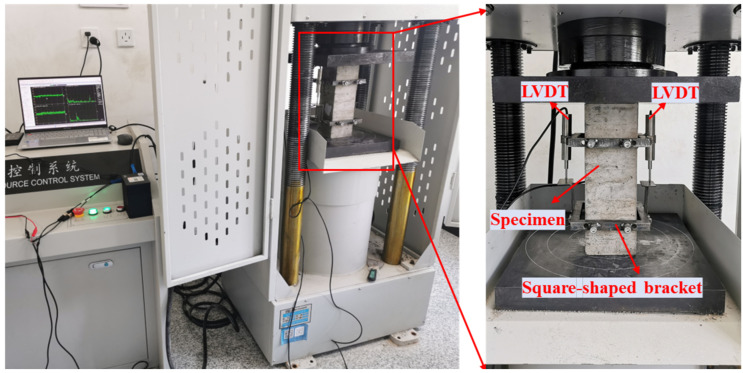
Test equipment.

The experimental setup is shown in Figure 4. The experimental procedure followed the method by Xiao et al. [34]. Axial compression tests were conducted using an electro-hydraulic servo testing machine under displacement-controlled loading at a rate of 0.2 mm/min. The axial load was automatically recorded by the testing system during loading, while axial deformation was simultaneously measured using two LVDT sensors mounted at both sides of the specimen. Stress–strain curves for prismatic specimens were recorded. The peak load calculated axial compressive strength, and the slope of the line from the origin to 40% of the peak stress determined the modulus of elasticity.

In addition, calculations were performed for strength reduction and elastic modulus reduction. The definitions of strength reduction and elastic modulus reduction are given by Equation (2) and Equation (3), respectively. Prior to the formal test [35], a pre-compression test was conducted to prevent the test results from being affected by leaving a gap between the test machine’s indenter and the specimen.

(2) $${∆}_{c}=\frac{F_{0}-F_{i}}{F_{0}}$$

where $F_{i}$ is the strength of RAC with different porosities or pore sizes, and $F_{0}$ denotes the strength of RAC without prefabricated pores ($P_{\mathrm{pore}}=0$).

(3) $${∆}_{E}=\frac{E_{0}-E_{i}}{E_{0}}$$

where $E_{i}$ represents the elastic modulus of RAC with different porosities or pore sizes, and $E_{0}$ denotes the elastic modulus of RAC without prefabricated pores ($P_{\mathrm{pore}}=0$).

### 2.3. High-Energy X-Ray CT Scanning

**Figure 5:**
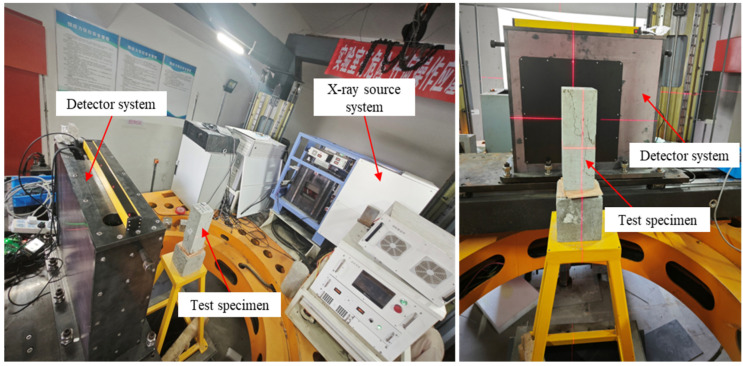
High-energy X-ray CT scanning process.

To further investigate the influence of pores on crack development in RAC under loading, the specimen after axial compression was scanned using a self-developed high-energy X-ray CT (Figure 5). The equipment can be switched between energy levels of 6 MeV and 9 MeV, thus enabling the scanning of large-sized specimens. In addition, considering the wide variety of specimens, a specimen with a pore diameter of 2–3 mm and a porosity of 8% was selected for scanning, based on considerations of crack development and the scanning resolution of the high-energy X-ray CT system. The scanning parameters for this equipment are provided in Table 3. During the scanning process, the specimen remained stationary while the detector and X-ray source performed a full 360° scan around it.

## 3. Results

### 3.1. Damage Patterns of RAC with Prefabricated Pores

**Figure 6:**
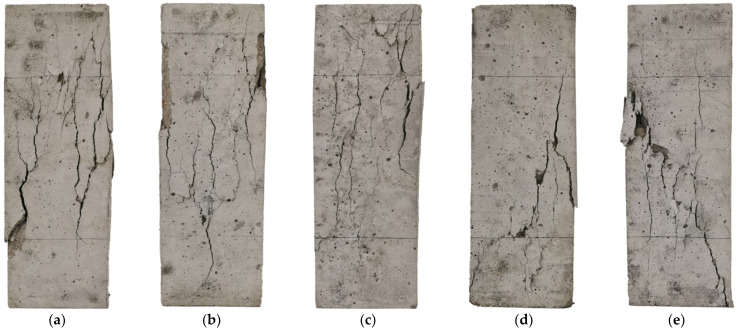
Damage patterns of RAC specimens with various pore sizes ($P_{\mathrm{pore}}=8\%$): (**a**) 0.3–0.5 mm; (**b**) 0.5–1 mm; (**c**) 1–2 mm; (**d**) 2–3 mm; (**e**) 3–5 mm.

Figure 6 shows the damage morphology of RAC with initial pores at different pore sizes under axial compressive loading. It can be observed that smaller pore sizes result in a higher number of surface cracks in RAC, and there are some fine and dense cracks. Conversely, with larger pore sizes, the number of surface cracks decreases, and penetrating diagonal cracks are more likely to form when the specimen is damaged. This is due to the presence of pores, which cause stress concentrations in both the pores and adjacent weak zones, thereby becoming potential crack initiation sites. When the porosity is the same, large pores are larger in size but fewer in number compared to small pores. When the specimen is subjected to external loading to produce cracks and propagate inside the specimen, the existence of large pores makes the cracks propagate with less resistance, making it more likely for cracks to connect between the pores. As a result, the overall number of cracks decreases, which makes it more likely to form a through-crack and ultimately leading to the failure of the RAC [36].

### 3.2. Effect of Porosity on Macromechanical Properties of RAC

**Figure 7:**
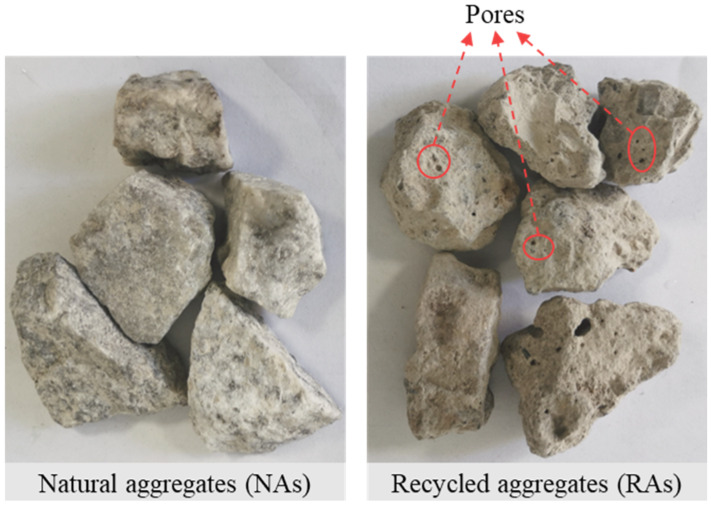
Comparison between NAs and RAs.

**Figure 8:**
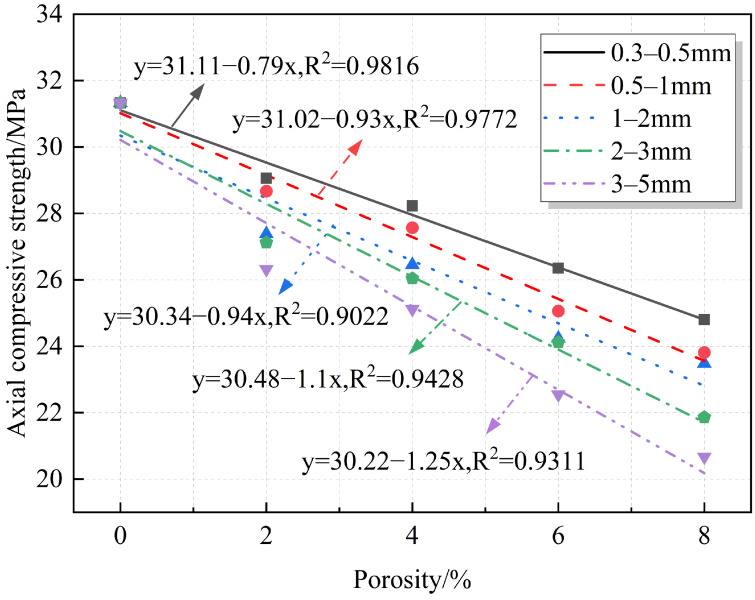
Fitted relationship between porosity and axial compressive strength.

**Figure 9:**
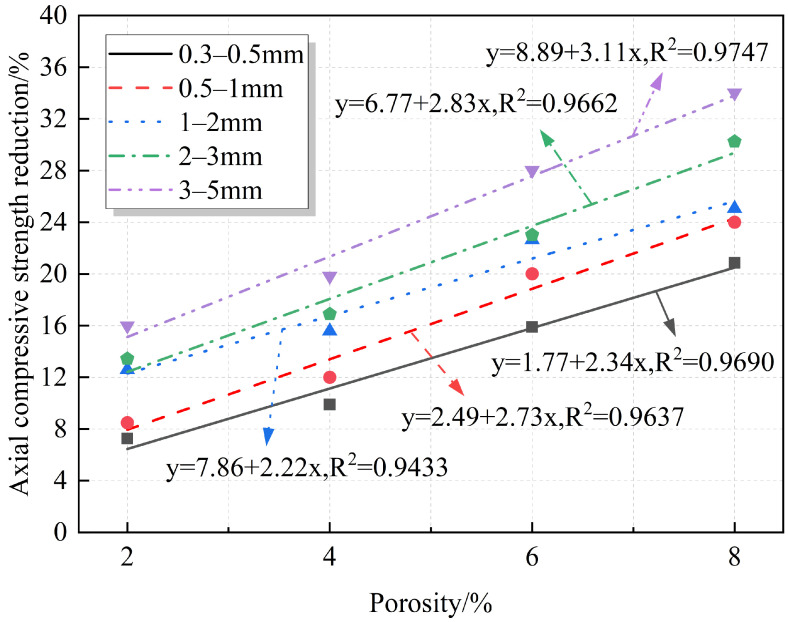
Fitted relationship between porosity and axial compressive strength reduction.

Axial compressive strength is one of the most intuitive mechanical indicators to study the degree of destruction of RAC by porosity. Currently, many studies quantitatively analyze the relationship between strength and porosity in concrete materials. Most of these studies propose prediction formulas based on semi-empirical equations, such as linear [17,37], exponential [38], logarithmic [15], and power functions [39]. However, unlike ordinary concrete, RAC is special in that it is made from RAs that partially or completely replace NAs. Figure 7 shows the comparison between RAs and NAs. It can be observed that the surfaces of the RAs are not only covered with a layer of old mortar but also contain natural pores. From an experimental perspective, developing an empirical formula that correlates porosity with the strength of RAC is crucial for its further application in construction projects. Therefore, the relationship between strength, strength reduction, and porosity of RAC was quantitatively analyzed. The fitting results are shown in Figure 8 and Figure 9. The fitting equations indicate that there is a linear relationship between the strength, strength reduction, and porosity of RAC. As shown, under the same pore sizes, the axial compressive strength decreases with the increase in the porosity, while strength reduction increases, reaching a maximum value of 34.02%.

The elastic modulus measures the ability of a material to resist elastic deformation and is strongly correlated with both the elastic modulus and surface characteristics of the aggregates [40]. Therefore, due to the porous properties of RAs, the elastic modulus of RAC specimens is affected not only by the internal porosity of the specimen but also by the RAs. Figure 7 shows that pores of different sizes are distributed on the crushed RAs from the actual project. Therefore, it is necessary to study the relationship between the elastic modulus of RAC and porosity from an experimental perspective.

**Figure 10:**
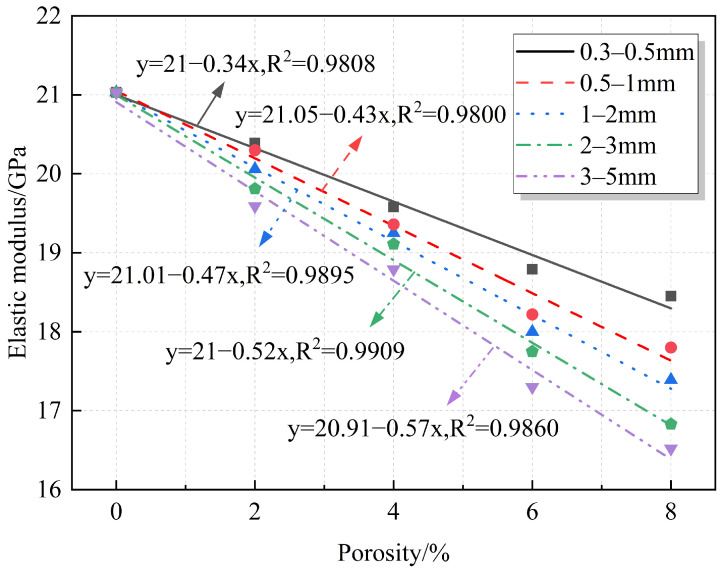
Fitting relationship between porosity and elastic modulus.

**Figure 11:**
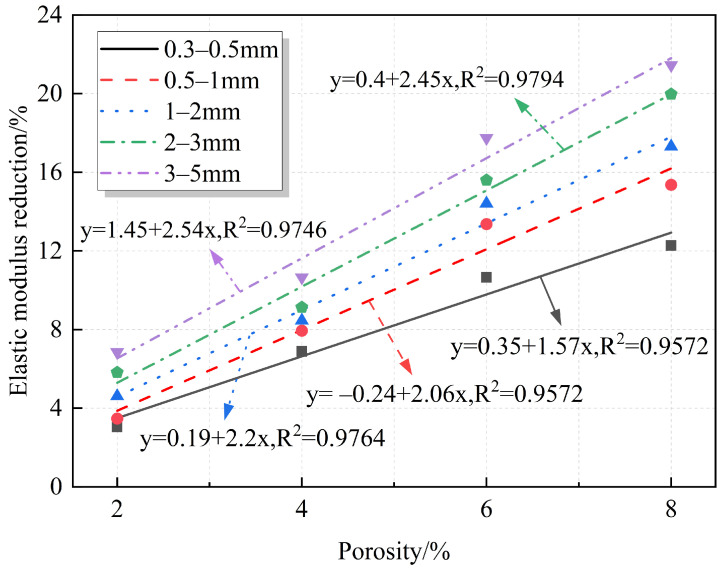
Fitting relationship between porosity and elastic modulus reduction.

The relationships between elastic modulus, modulus reduction, and porosity are quantified and plotted in Figure 10 and Figure 11, respectively. The elastic modulus and elastic modulus reduction exhibit a linear relationship with porosity. The figures show that the elastic modulus gradually decreases with increasing porosity, with the reduction reaching a maximum of 21.45%. This is because, despite the porous surface of RAs, their elastic modulus remains high compared to that of the prefabricated pores. The elastic modulus of the prefabricated pores, which are composed of air, is very small or even almost negligible. Therefore, the introduction of prefabricated pores weakens the mortar properties and reduces the overall stiffness of the specimen.

Within the porosity range studied in this research (2–8%), the prefabricated pores are predominantly isolated and randomly distributed, without forming interconnected large pores or macroscopic crack networks. Therefore, the weakening effect of porosity on mechanical properties is uniform and gradual, rather than leading to sudden failure. Consequently, macroscopic mechanical parameters such as axial compressive strength and elastic modulus exhibit an approximately linear decreasing trend with increasing porosity.

### 3.3. Effect of Pore Size on Macromechanical Properties of RAC

**Figure 12:**
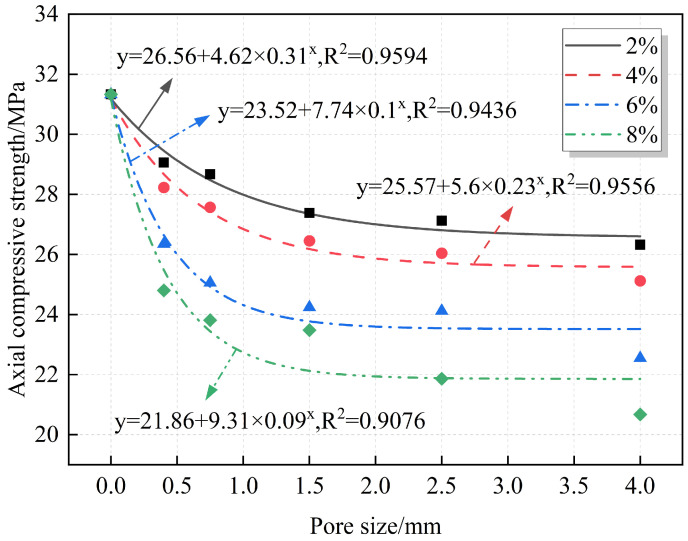
Fitting relationship between pore size and axial compressive strength.

**Figure 13:**
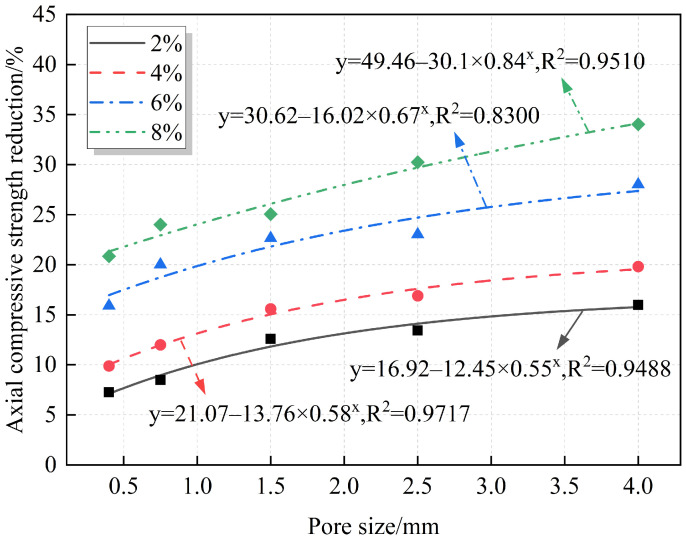
Fitting relationship between pore size and axial compressive strength reduction.

A quantitative analysis of the relationships between pore size and compressive strength and strength reduction is provided, with the results summarized in Figure 12 and Figure 13. At the same porosity, the strength decreases gradually with increasing pore size, and the strength reduction increases accordingly. The fitting equations reveal an exponential relationship between pore size and both axial compressive strength and strength reduction. From Figure 13, for the same porosity, the strength reduction increases with the increase in pore size, but the increase gradually slows down. This indicates that the compressive strength is more sensitive to small pore sizes, and the decrease in compressive strength is more obvious in the range of small pore sizes. For example, when the porosity is 8% and the pore sizes are 0.3–0.5 mm and 0.5–1 mm, the decrease in the compressive strength is 20.85% and 24%, respectively. When the pore size is 3–5 mm, the strength reduction is 34.02%. At this point, the average pore size increased by 10 times and 5.33 times, respectively, while the compressive strength reduction only increased by 1.63 times and 1.42 times, respectively. The strength reduction does not show the same proportion with the increase in pore size increase and a large difference between the two. This may be because, at the same porosity, the smaller the pore size, the larger the number of pores, which are more likely to be distributed in the weak zones between the mortar and the aggregate, or at the interfaces between the old and new mortar, resulting in a more sensitive change of the strength at smaller pore sizes.

**Figure 14:**
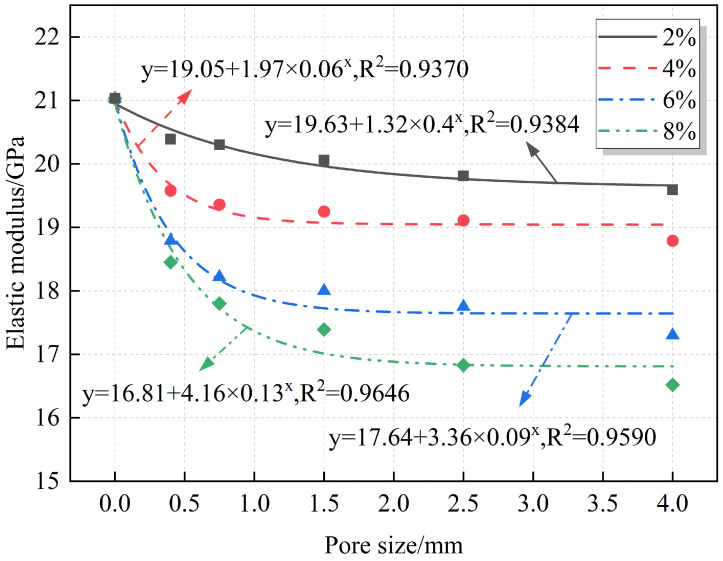
Fitting relationship between pore size and elastic modulus.

**Figure 15:**
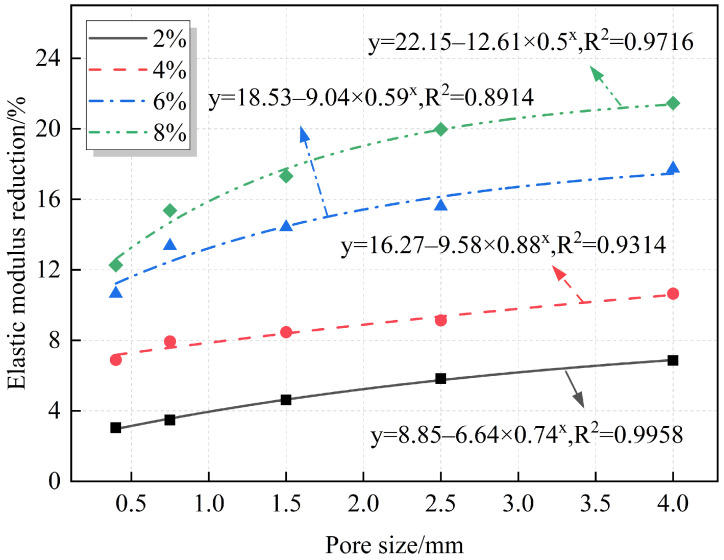
Fitting relationship between pore size and elastic modulus reduction.

A quantitative analysis of the relationships between the elastic modulus, modulus reduction, and pore size is summarized in Figure 14 and Figure 15. The fitting results indicate that both the elastic modulus and modulus reduction follow an exponential relationship with pore size. Under the same porosity, the elastic modulus decreases gradually as pore size increases, while the modulus reduction increases with pore size.

The relationship between pore size and mechanical properties exhibits a distinct nonlinear characteristic. This nonlinear behavior primarily arises from the enhanced local stress concentration effect at pore edges. As pore size increases, stress concentration in the pore edge regions intensifies, making cracks more likely to form and propagate along the pore edges. These phenomena reflect the typical nonlinear evolution mechanism of local damage, ultimately leading to an exponential decline in macroscopic mechanical properties such as axial compressive strength and elastic modulus with increasing pore size.

### 3.4. CT Scanning Results

**Figure 16:**
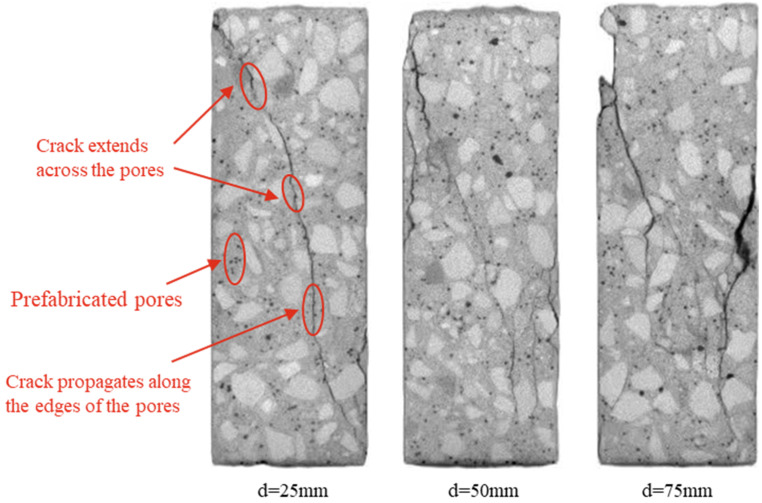
CT images of longitudinal sections at $P_{\mathrm{pore}}=8\%$ with pore size of 2–3 mm.

Figure 16 presents the scanned images of different longitudinal sections of RAC with prefabricated pores. In the figure, d represents the distance of any longitudinal section from the bottom surface of the specimen. The figure shows that the distribution of prefabricated pores in the different longitudinal sections is relatively uniform, confirming the effectiveness of the pore prefabrication method used in this study. Additionally, although the distribution of cracks varies across the longitudinal sections, the cracks in all sections predominantly propagate through the pores or along their edges. This suggests that the presence of pores not only creates weak points within the RAC but also significantly alters its internal stress distribution, thereby affecting the crack propagation path and accelerating the degradation of its macroscopic mechanical properties.

## 4. Correlation Analysis of Pore Structure and Macroscopic Mechanical Parameters Based on Gray Correlation Theory

According to Section 3.2 and Section 3.3, the effects of porosity and pore size on the macro-mechanical parameters were analyzed separately, and both have a strong influence on these parameters. However, it remains unclear which of the two exerts a greater influence. Gray correlation analysis (GCA) is a branch of gray system theory. GCA finds the degree of similarity or dissimilarity of the development trend of each influencing factor through quantitative methods, obtains the mathematical relationship between these factors, and identifies the most significant ones [41]. The advantage of GCA lies in its ability to accurately assess the correlation between factors using minimal data and simpler calculations [42]. Therefore, in this section, GCA is applied to analyze the degree of correlation between porosity, pore size, and macroscopic mechanical parameters, specifically focusing on the compressive strength and the elastic modulus, and the degree of influence is quantitatively evaluated. The calculation steps for GCA are as follows:

(1) We identify the reference and comparison sequences.

The data sequence reflecting the behavioral characteristics of the system is defined as the reference sequence $X_{i}$, which can be represented by Equation (4).

(4) $$X_{i}=\left\{X_{i}\left(k\right)\left|k=1,2,3\cdots n,\;i=1,2,3\cdots g\right.\right\}$$

The sequence composed of factors affecting the behavioral characteristics of the system is defined as the comparison sequence $Y_{o}$, which can be represented by Equation (5).

(5) $$Y_{o}=\left\{Y_{o}\left(k\right)\left|k=1,2,3\cdots n,\;o=1,2,3\cdots \right.m\right\}$$

(2) The initial value transformation method [43] is used to obtain $x_{i}$ and $y_{o}$. We nondimensionalize the reference sequence $X_{i}$ and comparison sequence $Y_{0}$, respectively, as shown in Equations (6) and (7).

(6) $$x_{i}=\frac{X_{i}}{X_{i}\left(1\right)},\;i=1,2,3\cdots g$$

(7) $$y_{o}=\frac{Y_{o}}{Y_{o}\left(1\right)},\;o=1,2,3\cdots m$$

(3) We calculate the absolute difference $∆\varepsilon_{o}\left(k\right)$ between $x_{i}$ and $y_{o}$ as shown in Equation (8).

(8) $$∆\varepsilon_{o}\left(k\right)=\left|x_{i}\left(k\right)-y_{o}\left(k\right)\right|,\left(k=1,\cdots ,n;i=1,\cdots ,g\right)$$

(4) We calculate the gray correlation coefficient $\varepsilon_{o}\left(k\right)$ as shown in Equation (9).

(9) $$\varepsilon_{o}\left(k\right)=\frac{\underset{o}{\min}\underset{k}{\min}\left|x_{i}\left(k\right)-y_{o}\left(k\right)\right|+\alpha \times \underset{o}{\max}\underset{k}{\max}\left|x_{i}\left(k\right)-y_{o}\left(k\right)\right|}{∆\varepsilon_{o}\left(k\right)+\alpha \times \underset{o}{\max}\underset{k}{\max}\left|x_{i}\left(k\right)-y_{o}\left(k\right)\right|},\left(k=1,\cdots ,n\right)$$

where α is the resolution coefficient used to weaken the two levels. If the maximum difference is too large to make the correlation coefficient distortion, its value is generally taken as 0.1~0.5. This paper takes the value of 0.5. $\underset{o}{\min}\underset{k}{\min}\left|x_{i}\left(k\right)-y_{o}\left(k\right)\right|$ is the second-order minimum absolute difference between the reference sequence and the comparison sequence, and $\underset{o}{\max}\underset{k}{\max}\left|x_{i}\left(k\right)-y_{o}\left(k\right)\right|$ is the second-order maximum absolute difference between the reference sequence and the comparison sequence.

(5) We calculate the degree of gray correlation $\delta_{o}$ as shown in Equation (10).

(10) $$\delta_{o}=\frac{1}{n}\sum_{k=1}^{n}\varepsilon_{o}\left(k\right)$$

where $\delta_{o}\in \left(0,\right.\left.1\right]$; when $0<\delta_{o}\leq 1$, $Y_{0}$ is correlated with $X_{i}$; when $\delta_{o}=1$, $Y_{0}$ is strictly correlated with $X_{i}$; when $\delta_{o}=0$, $Y_{0}$ is not correlated with $X_{i}$. $\delta_{o}$ is closer to 1 the better the correlation between $Y_{0}$ and $X_{i}$.

**Figure 17:**
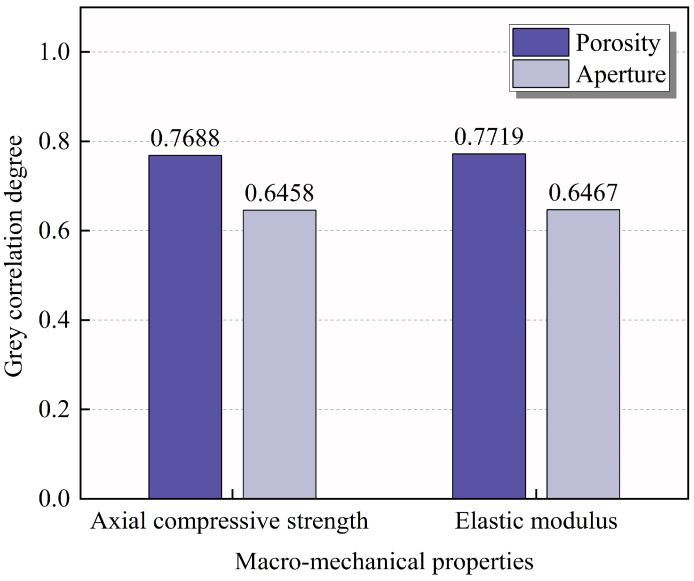
Grey correlation degree of pore structure properties with compressive strength and elastic modulus.

Based on the above theory, the compressive strength and elastic modulus are taken as the reference sequence, respectively. The strength is denoted as $X_{1}$, and the elastic modulus is denoted as $X_{2}$. Porosity and pore size are taken as the comparison sequences, with porosity denoted as $Y_{1}$ and pore size as $Y_{2}$. The gray correlation of porosity and pore size on the macro-mechanical parameters is illustrated in Figure 17. The gray correlation of porosity and pore size with axial compressive strength are 0.7688 and 0.6458, respectively, while the gray correlation with the elastic modulus are 0.7719 and 0.6467, respectively. Porosity and pore size have a significant effect on both strength and elastic modulus. While most studies [44,45,46] focus on the weakening effect of porosity on the mechanical properties of materials, the results of this study confirm the influence of pore size. Therefore, in addition to considering total porosity, the influence of pore size on the macroscopic mechanical properties should also receive attention. In addition, the influence of porosity on both the strength and modulus of elasticity of RAC was significantly greater than that of pore size.

## 5. Combined Effect of Porosity and Pore Size

The response of the macroscopic mechanical properties reflect the degree of deterioration within the concrete material [47]. Damage mechanics theory can be applied to investigate the macro-mechanical degradation of concrete, which results from the formation and growth of internal defects under external forces. The damage variable D can be used to describe the effect of internal cracks, pores, and other damage on the macro-mechanical parameters, thereby quantitatively characterizing the degree of material deterioration. To further analyze the combined effect of porosity and pore size, this section introduces the damage variable D, $D\in \left[0,1\right]$. The variable D is used to characterize the degree of damage to the macroscopic mechanical parameters of the material at different porosities and pore sizes. When D = 1, the material is fully damaged. When D = 0, the material is undamaged. The damage degree of compressive strength $D_{c}$ and elastic modulus $D_{e}$ are illustrated in Equations (11) and (12), respectively.

(11) $$D_{c}=1-\frac{\tilde{f}}{f_{0}}$$

where $\tilde{f}$ represents the strength of RAC with prefabricated pores, and $f_{0}$ represents the strength of the RAC without prefabricated pores.

(12) $$D_{e}=1-\frac{\tilde{E}}{E_{0}}$$

where $\tilde{E}$ represents the elastic modulus of RAC with prefabricated pores, and $E_{0}$ represents the elastic modulus of RAC without prefabricated pores.

**Figure 18:**
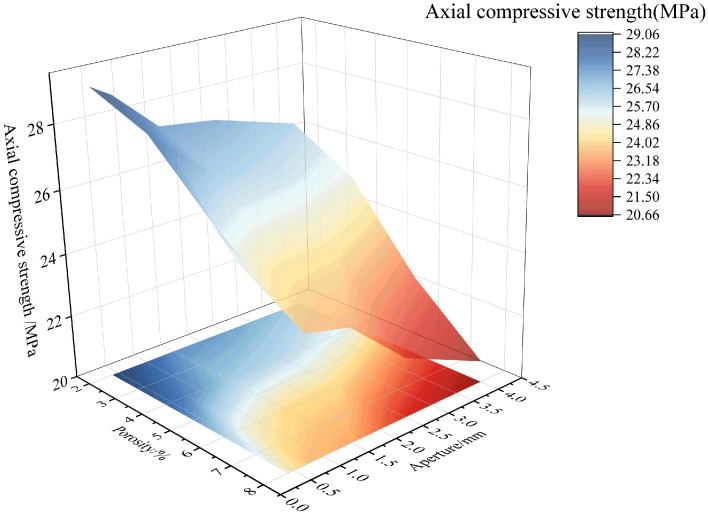
Combined effect of porosity and pore size on axial compressive strength.

**Figure 19:**
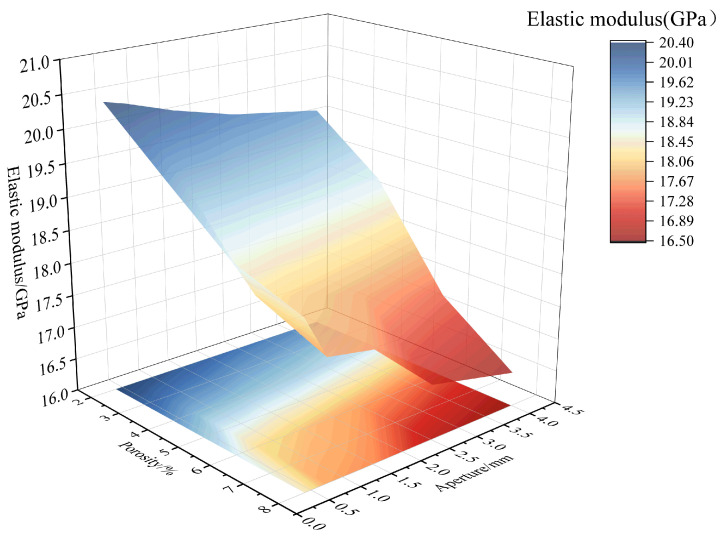
Combined effect of porosity and pore size on elastic modulus.

**Figure 20:**
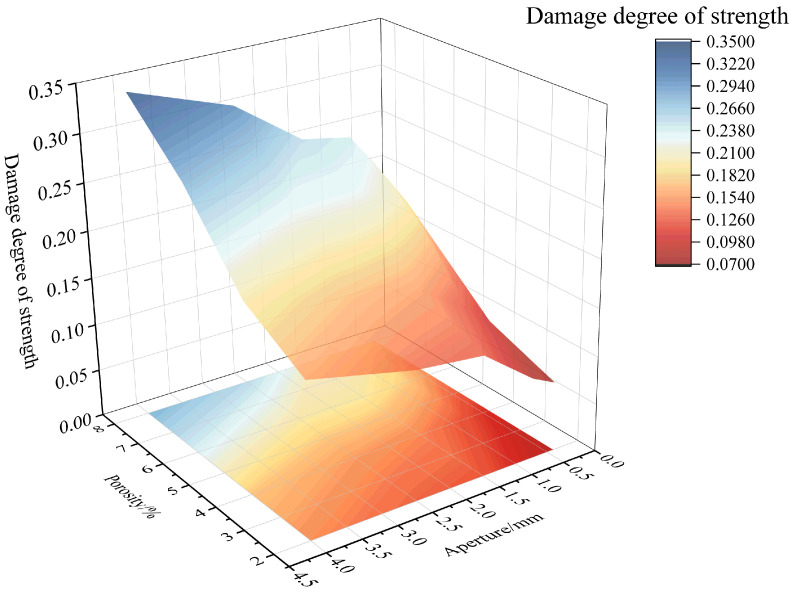
Combined effect of porosity and pore size on damage degree of strength.

**Figure 21:**
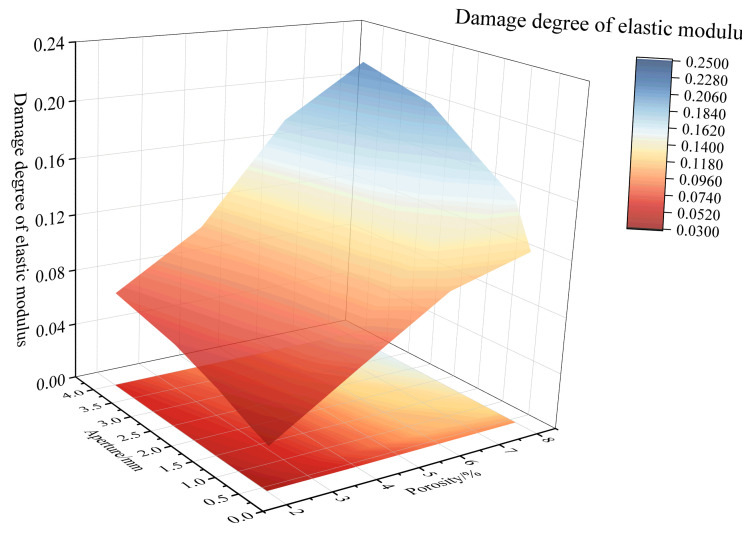
Combined effect of porosity and pore size on damage degree of elastic modulus.

To further analyze the combined effect of porosity and pore size on the mechanical properties, the changes in compressive strength and elastic modulus under the influence of both factors are shown in Figure 18 and Figure 19, respectively. Additionally, the relationships between the combined effect of porosity and pore size on the strength and elastic modulus damage degree of RAC are plotted according to Equations (11) and (12), as shown in Figure 20 and Figure 21. According to Figure 18 and Figure 20, it can be found that when considering the combined effects of pore size and porosity, the axial compressive strength reaches its minimum value when both porosity and pore size are high. In this case, the compressive strength decreases most significantly, and the degree of damage to strength reaches its maximum value of 0.3403. The simultaneous increase in both porosity and pore size significantly negatively affects compressive strength. Furthermore, the degree of compressive strength damage is higher for the combination of small pore size and high porosity than for the combination of large pore size and low porosity. For example, when the pore size is 0.3–0.5 mm and the porosity is 8%, the damage degree of compressive strength is 0.2084. In contrast, when the pore size is 3–5 mm and the porosity is 2%, the damage degree of compressive strength is 0.1599.

Observing Figure 19 and Figure 21, considering the combined effect of porosity and pore size, as the porosity and pore size increase, the decrease in the elastic modulus becomes more significant, and the degree of damage to the elastic modulus increases. At the combination of high porosity and large pore size, the elastic modulus reaches its minimum value, and the damage degree of the elastic modulus reaches its maximum value of 0.2145. Furthermore, the elastic modulus damage degree is higher for the combination of small pore size and high porosity than for the combination of large pore size and low porosity. For example, when the pore size is 0.3–0.5 mm and the porosity is 8%, the elastic modulus damage degree is 0.1227. In contrast, when the pore size is 3–5 mm and the porosity is 2%, the elastic modulus damage degree is 0.0685.

## 6. Conclusions

This study investigates the influences of porosity and pore size on the mechanical properties of RAC through axial compression tests. A quantitative analysis was conducted to explore the relationships between porosity, pore size, and macro-mechanical parameters. The crack development influenced by pores was analyzed using high-energy X-ray CT. The influences of porosity and pore size on these macro-mechanical properties were evaluated using gray correlation analysis. Additionally, the combined effect of porosity and pore size on the macro-mechanical performance was assessed in terms of the degree of damage. The key findings are as follows:

(1) The strength decreases as porosity and pore size increase, while the strength reduction increases. The strength reduction reaches a maximum of 34.02% when the porosity is 8% and the pore size is 3–5 mm. In addition, both the axial compressive strength and its reduction exhibit a linear relationship with the porosity and an exponential relationship with the pore size.
(2) The elastic modulus of RAC decreases as porosity and pore size increase, while the reduction in elastic modulus increases. The elastic modulus reduction reaches a maximum of 21.45% when the porosity is 8% and the pore size is 3–5 mm. In addition, both the elastic modulus and its reduction exhibit a linear relationship with the porosity and an exponential relationship with the pore size.
(3) CT images reveal that cracks within the specimen predominantly propagate through the pores or along their edges.
(4) Gray correlation analysis reveals that both porosity and pore size significantly influence the strength and elastic modulus. The effects of porosity on strength and elastic modulus are notably greater than those of pore size.
(5) When the porosity is 8% and the pore size is 3–5 mm, i.e., under the combined effect of high porosity and large pore size, the degrees of damage to both the strength and elastic modulus of RAC reach their maximum values, which are 0.3403 and 0.2145, respectively. The simultaneous increase in both porosity and pore size has a significant negative impact on the macro-mechanical properties. Furthermore, the damage to strength and elastic modulus is greater with the combination of small pore size and high porosity than with the combination of large pore size and low porosity.

## Figures and Tables

**Table 1:** Physical properties of RAs.

Particle Size/mm	Apparent Density/kg·m^−3^	Power Content/%	Water Absorption/%	Crushing Value/%
5–25	2500	0.5	2.6	10

**Table 2:** Mix proportion of RAC (kg/m^3^).

Sand	Recycled Coarse Aggregate	Cement	Water	Water-Reducing Agent
685	1019	400	223	1.68

**Table 3:** High energy X-ray CT scanning parameters.

Direct Current Voltage	Direct Current	Electron Gun Voltage	Average Magnetic Current	Average Gun Current	Resolution
455.65 V	4.06 A	264.4 V	49.9 mA	567 μA	100 μm

## Data Availability

The original contributions presented in the study are included in the article. Further inquiries can be directed to the corresponding author.
